# TGFβ1 and HGF regulate CTGF expression in human atrial fibroblasts and are involved in atrial remodelling in patients with rheumatic heart disease

**DOI:** 10.1111/jcmm.14165

**Published:** 2019-01-29

**Authors:** Jian‐Quan Chen, Yan‐Song Guo, Qian Chen, Xian‐Lu Cheng, Guo‐Jian Xiang, Mei‐Yan Chen, Hong‐Lin Wu, Qi‐Lei Huang, Peng‐Li Zhu, Jian‐Cheng Zhang

**Affiliations:** ^1^ Provincial Clinical Medicine College of Fujian Medical University Fuzhou PR China; ^2^ Department of Cardiology Fujian Provincial Hospital Fuzhou PR China; ^3^ Depatement of Critical Care Medicine Division Four Fujian Provincial Hospital Fuzhou PR China; ^4^ Depatement of Cardiology Nanping First Hospital Affiliated to Fujian Medical University Nanping PR China; ^5^ Depatement of Anesthesiology Division Two Fujian Provincial Hospital Fuzhou PR China; ^6^ Department of Geriatric Medicine Fujian Provincial Hospital Fujian Provincial Center for Geriatrics Fuzhou PR China

**Keywords:** atrial fibrillation, connective tissue growth factor, fibroblasts, hepatocyte growth factor, transforming growth factor β1

## Abstract

**Objective:**

This study aimed to investigate the effects of transforming growth factor β1 (TGF β1) and hepatocyte growth factor (HGF) on the expression of connective tissue growth factor (CTGF) in human atrial fibroblasts, and to explore the relationship of these factors in atrial fibrosis and atrial anatomical remodelling (AAR) of patients with atrial fibrillation (AF).

**Methods:**

Fresh right auricular appendix tissue of 20 patients with rheumatic heart disease undergoing valve replacement surgery was collected during surgeries, 10 patients had sinus rhythm(SR), and 10 patients had chronic atrial fibrillation (CAF). Atrial fibroblasts were then cultured from the tissues with differential attachment technique and treated with either TGFβ1 (10 ng/mL) or HGF (100 ng/mL). CTGF mRNA levels were measured by RT‐PCR, and CTGF protein content was determined using immunofluorescence and Western blotting assays.

**Results:**

CAF group had higher left atrial diameters (LADs) and higher CTGF mRNA expression in atrial fibroblasts compared with SR group. The CTGF protein content in CAF group was higher than that of SR group and positively correlated with LAD and AF duration. After CAF group was treated with TGFβ1, CTGF mRNA and protein expression were significantly down‐regulated, whereas when treated with HGF, expression was up‐regulated compared with SR group.

**Conclusions:**

Increased CTGF expression was associated with enlarged LAD, atrial fibrosis and AAR in patients with AF. TGFβ1 and HGF regulate CTGF expression in human atrial fibroblasts with up‐regulation of mRNA and down‐regulation of protein, therefore, either promote or inhibit atrial fibrosis, which could be related to the incidence and persistence of AF.

## 1 INTRODUCTION

Atrial fibrillation (AF) is the most common arrhythmia^1^ and has high morbidity and mortality. The main complications are thromboembolic events, stroke and heart failure. Rheumatic heart disease (RHD) is one cause of AF, for which a mechanism is not fully understood. AF occurrence and persistence are related to atrial anatomical remodelling (AAR). Myocardial fibrosis (MF) is one of the main pathological changes of the myocardium, which reflects diseases prognosis. AF promotes electrical and structural remodelling of the atrial tissue, forms ‘matrices’, increases AF relapse probabilities and prolongs AF durations.^2^ Atrial fibrosis is the hallmark of AF‐dependent structural remodelling.^3^ Studies have shown that atrial fibrosis is significantly increased in patients with AF.^4^, ^5^ MF is a huge global burden, and new treatment options are greatly needed.^6^

MF progression is controlled by various factors, including the renin‐angiotensin‐aldosterone system, chemokines, cytokines and growth factors.^7^, ^8^ Fibrosis is mainly characterized by the deposition of extracellular matrix (ECM), and fibrosis‐associated biomarker detection provides insight into cardiac ECM remodelling.

Transforming growth factor‐β1 (TGFβ1) is one of the most potent cytokines and is involved in many biological processes including fibrosis, tissue repair, anti‐inflammatory responses, hypertrophy and atherosclerosis.^9^ In the initial stages of myocardial remodelling, TGFβ1 promotes fibroblast activation and induces ECM protein production including collagen and fibronectin. Connective tissue growth factor (CTGF) is a multi‐domain stromal cell protein that mediates fibrosis in various tissues and is a key downstream mediator of the TGFβ1 signalling pathway in fibroblasts.^10^, ^11^ Studies have shown that TGFβ1 enhances the activity and expression of the CTGF promoter in cardiac fibroblasts.^12^ CTGF is an effective pro‐fibrotic factor under various pathophysiological conditions including AF.^13^ Using a mouse model of myocardial infarction, it was confirmed that TGFβ and CTGF were expressed in scar tissue fibroblasts.^14^ The increased content of CTGF in the left atrial tissue was associated with enhanced fibrosis in AF patients. However, hepatocyte growth factor (HGF) has an anti‐TGFβ1 effect and has been shown to reverse the fibrotic process in animal models. HGF significantly reduces TGFβ1‐induced CTGF production in tubulointerstitial fibroblasts^15^ and antagonizes MF by inhibiting the endothelial‐mesenchymal transition.^16^

TGFβ1 and HGF promote and inhibit MF, respectively, and these regulatory effects may be related to the molecular mechanisms of MF. As TGFβ1 has multiple functions in human physiology, blocking its effects could result in adverse outcome.^17^ CTGF, which is a mutual TGFβ1 and HGF downstream signalling factor, acts more specifically on connective tissue,^18^ promoting fibrosis and ECM production. Data suggest that although enhanced CTGF expression is closely linked to fibrosis, CTGF alone is insufficient to promote the progression of fibrosis in pathophysiological environments. It is assumed that several factors need to be combined to turn CTGF into a pro‐fibrotic and 'pro‐arrhythmic' factor. And, in another study, CTGF was proposed as a new target against renal fibrosis.^19^ This study investigated the ‘network’ of TGFβ1, HGF and CTGF, with the expectation that CTGF will be a new therapeutic target of MF.

To study the occurrence and persistence of AF at the cellular and molecular level, our laboratory developed methods to isolate and culture human atrial fibroblasts.^20^ In this study, atrial fibroblasts from RHD patients were selected as the target cells. The effects of HGF and TGFβ1 on CTGF secretion in human atrial fibroblasts were investigated, and the relationship between CTGF and left atrial diameter (LAD), and duration of AF episodes was evaluated. We emphasized potential therapeutic targets and the significance of these targets in the clinical treatment of AF. The results of this study will be beneficial for those who want to understand the key molecular mechanisms of AAR and atrial fibrosis in patients with AF and provide evidence for the prevention and treatment of AF.

## 2 MATERIALS AND METHODS

### 2.1 General data

Twenty patients with RHD who underwent valve replacement surgery in Fujian Provincial Hospital from January 2008 to July 2012 were enrolled, including nine males and 11 females. Patients were divided into sinus rhythm (SR) and chronic atrial fibrillation (CAF) groups, with 10 patients in each group. According to the 2016 European Society of Cardiology Guidelines for the Management of Atrial Fibrillation,^21^ AF episodes lasting longer than 7 days or 1year were referred to as CAF or long‐term CAF, respectively. In this study, the CAF group was defined as patients with AF episodes lasting longer than 6 months. AF was diagnosed with an electrocardiogram or a dynamic electrocardiogram. Clinical data from the electrocardiogram and echocardiography were collected. Informed consent was provided by the patients of this study and approved by the Ethics Committee of the Fujian Provincial Hospital.

### 2.2 Exclusion criteria

Patients with severe heart failure, hypertension, coronary atherosclerotic heart disease, chronic pulmonary heart disease, ischemic or non‐ischemic cardiomyopathy, hyperthyroidism, and recent (five drug half‐lives) intake of angiotensin‐converting enzyme and angiotensin II receptor inhibitors, aldosterone receptor antagonists and statin lipid‐lowering drugs were excluded.

### 2.3 Experimental reagents and devices

An Olympus IMT221 inverted phase contrast microscope (Olympus, Tokyo, Japan), a DMiRB fluorescence inverted microscope (Leica, Wetzlar, Germany), a 3336 CO2 incubator (Forma, Watertown, MA, USA), DMEM high glucose medium (Gibco, Grand Island, NY, USA), foetal bovine serum (Hyclone, South Logan, UT, USA), antimouse cy3 and anti‐rabbit FITC immunofluorescence staining kits, BCA protein Quantification kits (Beyotime Biotechnology), the V2258 mouse vimentin antibody, the A2547 mouse α smooth muscle actin (α‐SMA) monoclonal antibody (Sigma, Saint Louis, MO, USA), a DYC‐p31D electrophoresis apparatus (Beijing Liuyi Instrument Factory, Beijing, China), a Gene Amp 9700 PCR amplification instrument (ThermoFisher, Waltham, MA, USA), an RT‐PCR A3500 kit (Promega), a NanoDrop ND‐1000 UV spectrophotometer (NanoDrop, Wilmington, DE, USA), an ImageMaster VDS‐CL gel analyser, MiniVE protein electrophoresis equipment, TE22 electrophoresis equipment (Amersham Biosciences, Staffanstorp, Sweden), TRIzol(Invitrogen, Carlsbad, CA, USA), human recombinant HGF, TGFβ1 (PeproTech, Rocky Hill, NJ, USA), SC‐14939 goat anti‐human CTGF antibody (SANTA CRUZ), rabbit anti‐goat IgG antibody labelled with horseradish peroxidase and a goat anti‐goat Mouse IgG antibody (Beijing Zhongzhan Jinqiao Biotechnology Co., Ltd., Beijing, China) were used in the study.

### 2.4 Isolation, culture and identification of human atrial fibroblasts and experimental grouping

Fresh aseptic right atrial appendix tissue (20 mg) was collected during valve replacement surgeries of the RHD patients. Samples were kept in large EP tubes containing aseptic PBS (placed on ice) and transported to the laboratory within 15 minutes of tissue acquisition. Atrial fibroblasts were isolated using enzyme digestion and differential adherence methods previously developed by our laboratory.^20^ Cells were cultured in DMEM containing 20% foetal bovine serum at 37°C in a 5% CO2 incubator. Cell growth was observed with an inverted microscope, and atrial fibroblasts were passaged after reaching 80% confluence. At the third passage, fibroblasts were identified by α‐SMA and vimentin immunofluorescent antibody staining.^20^ The second or third passages of cells were used in the following studies. Fibroblasts were divided into five groups: the ①SR group; the ②CAF group; ③ the TGFβ1 intervention group (10 ng/mL TGFβ1 was added to the CAF group cells); ④the HGF intervention group (100 ng/mL HGF was added to the CAF group cells); and ⑤ the TGFβ1+HGF intervention group (10 ng/mL TGFβ1 and 100 ng/mL HGF were added to the CAF group cells).

### 2.5 RT‐PCR

CTGF mRNA levels were determinated using RT‐PCR technology. Total RNA was extracted using the single‐step method with the TRIzol reagent (Invitrogen, USA). The first‐strand cDNA was synthesized by the two‐step method of the reverse transcription kit using 2 µL total RNA (with a final concentration of 1 μg/μL) and was used as the PCR template. The PCR products were synthesized and separated using 2% agarose gel electrophoresis, stained with the nucleic acid dye, GELVIEW, and scanned using a gel imaging system. The relative content of CTGF mRNA was determined by calculating the integral ratio of gray‐scale values of the CTGF gene and the internal reference GAPDH fragments. Primer sequences and amplified fragments were GAPDH: upstream 5'‐aga agg ctg ggg ctc att tg‐3' and downstream 5'‐Agg ggc cat cca cag tct tc‐3' cDNA, the fragment length was 258 bp; and CTGF: upstream 5' ‐CCAACTATGATTAGAGCCAACTG‐3', downstream 5'‐AGGCACAGGTCTTGATGA AC‐3' cDNA, the fragment length was 400 bp. The PCR reaction procedure was 35 cycles of pre‐denaturation at 94°C for 5 minutes, followed by denaturation at 94°C for 30 seconds, annealing at 60°C for 45 seconds, and extensions at 72°C for 1 minute, and extensions at 72°C for 10 minutes.

### 2.6 Immunofluorescence and Western blotting analysis

CTGF protein expression was detected using immunofluorescence and Western blot assays. One mL of a 2 × 10^6^ atrial fibroblast suspension (in DMEM medium containing 20% foetal bovine serum) was added to each well of a 6‐well plate and cultured for 24 hours. The media were then replaced with serum‐free DMEM, and the cells were incubated for another 24 hours. At this point, the culture media were discarded, and appropriate cytokines were added to the wells of the intervention groups (in DMEM culture medium containing 2% foetal bovine serum). Cells were cultured for 48 hours, and then the culture medium was discarded. Fibroblasts were fixed with 1 mL 4% paraformaldehyde for 30 minutes, washed with PBS buffer three times, and blocked with 1 mL blocking solution on a shaker for 60 minutes. The blocking solution was removed, and the primary goat anti‐human CTGF antibody (1:200) was added. The reaction system was incubated at 4°C overnight and washed three times (3‐5 minutes each time). Then, the fluorescence‐labelled secondary anti‐goat FITC antibody (1:100) was added, and samples were incubated for 1 hour on a shaker, washed three times, observed with a fluorescent microscope after sealing, and developed with FITC. Imagepro Plus software and the SigmaScan 4.0 system were used for analysing the immunofluorescence images to determine the cumulative integrated optical density (IOD).

Western blot extraction and SDS‐PAGE gel electrophoresis were performed according to the previously described method.^22^ Briefly, 2 mL of the cell suspensions were added to each well. After performing 10% SDS‐PAGE, the CTGF protein was electrophoretically transferred to a nitrocellulose (NC) membrane with a constant voltage. The NC membrane was successively incubated with primary antibodies (a mouse anti‐human GAPDH monoclonal antibody [1:500] and a goat anti‐human CTGF antibody [1:400]), and secondary horseradish peroxidase‐labelled goat antimouse IgG antibody (1:10000) overnight at 4°C. Then, samples were washed and incubated for 2 hours at room temperature, and then washed and stained. The NC film was attached to X‐ray film for exposure and development. ImageQuant TLV2003 software was used to analyse the gray‐scale values. The relative CTGF protein content was determined by calculating the ratio of the gray‐scale values of CTGF protein against the internal reference, GAPDH.

### 2.7 Statistical analysis

Excel 2010 and SPSS 21.0 were used to process and analyse data. The study data were expressed as mean ± standard deviation ($\overset{¯}{x}\pm s$), and the mean between the SR and CAF groups was compared using a bilateral *t*‐test. Means of the two groups were compared with the one‐way ANOVA and the least significant difference (LSD) test. The correlation between indicators was analysed with correlation and regression analysis. The results were considered statistically significant when *P* < 0.05.

## 3 RESULTS

### 3.1 General data

Table 1 demonstrates the baseline patient characteristics. No significant differences between the SR and CAF groups with respect to age (41.10 ± 5.22 vs 44.30 ± 5.25 years), the ratio of male patients (50% vs 40%) or left ventricular ejection fraction (LVEF) (54.68 ± 6.76% vs 51.83 ± 5.82%) were found. The duration of AF episodes in the CAF group was 55.74 ± 35.27 months, and the LAD measurements in the CAF group were significantly larger than that of the SR group (5.76 ± 1.37 vs 4.43 ± 0.77, *t* = 2.692, *P* < 0.05).

**Table 1:** Baseline characteristics of the study patients (N = 10)

Characteristics	Group SR	Group CAF
Male	5 (50%)	4 (40%)
Age (y)	41.10 ± 5.22	44.30 ± 5.25
Duration of AF (mo)	—	55.74 ± 35.27
LAD (cm)	4.43 ± 0.77	5.76 ± 1.37
LVEF (％)	54.68 ± 6.76	51.83 ± 5.82

### 3.2 Culture and identification of human atrial fibroblasts

**Figure 1:**
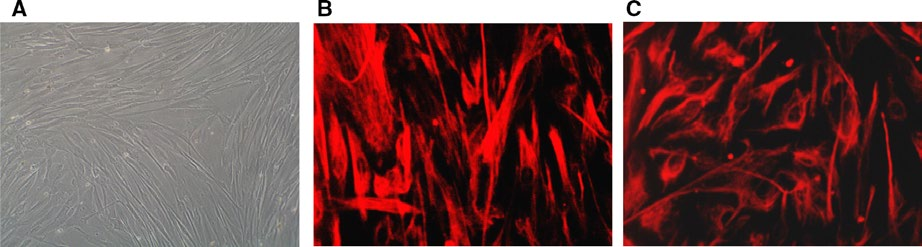
Human atrial fibroblasts (A) (observed with an inverted microscope ×250; Vimentin (B) and α‐SMA (C) immunostaining show positive staining of atrial fibroblasts ×400)

Figure 1A shows fibroblast morphology observed with a microscope. Fibroblasts were identified as myocardial fibroblasts (MFbs) using cytoplasmic vimentin and α‐SMA antibody immunofluorescent stains (Figures 1B,C). The cells were passaged and divided into the five groups.

### 3.3 The effects of TGFβ1 and HGF on CTGF mRNA expression in atrial fibroblasts

**Figure 2:**
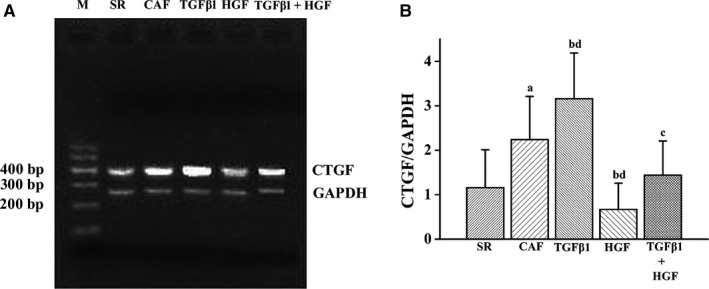
Connective tissue growth factor mRNA expression in human atrial fibroblasts as detected using RT‐PCR. CTGF mRNA expression is shown in A. M indicates the 100 base pair ladder marker; GAPDH, glyceraldehyde triphosphate dehydrogenase (258 bp); B is comparative histograms of CTGF mRNA expression in each experimental group (n = 10). In order of appearance: (A) the CAF group compared with the SR group (*P* < 0.01); (B) the TGFβ1 group compared with the CAF group (*P* < 0.01) and (D) with the TGFβ1+HGF group; (B) the HGF group compared with the CAF group (*P* < 0.01), and (D) with the TGFβ1 + HGF group (*P* < 0.01); (C) the TGFβ1 + HGF group compared with the CAF group (*P* < 0.05)

The positions of the amplified CTGF and GAPDH electrophoretic bands were 400 bp and 258 bp, which was consistent with the set lengths of the fragments (Figure 2A). The relative CTGF mRNA expression level was significantly increased in the CAF group compared with the SR group (*P* < 0.01). CTGF mRNA expression in the SR, CAF, TGFβ1, HGF and TGFβ1+HGF groups were 1.16 ± 0.85, 2.24 ± 0.97, 3.16 ± 1.03, 0.67 ± 0.59 and 1.44 ± 0.77, respectively (Figure 2B). Compared with the CAF group, CTGF mRNA expression was higher in the TGFβ1 group (*P* < 0.01) and lower in the TGFβ1+HGF group (*P* < 0.05) and the HGF group (*P* < 0.01). Compared with the TGFβ1+HGF group, CTGF mRNA expression was lower in the HGF group (*P* < 0.05) and higher in the TGFβ1 group (*P* < 0.01) (Figure 2B).

### 3.4 CTGF protein expression in myocardial atrial fibroblasts

**Figure 3:**
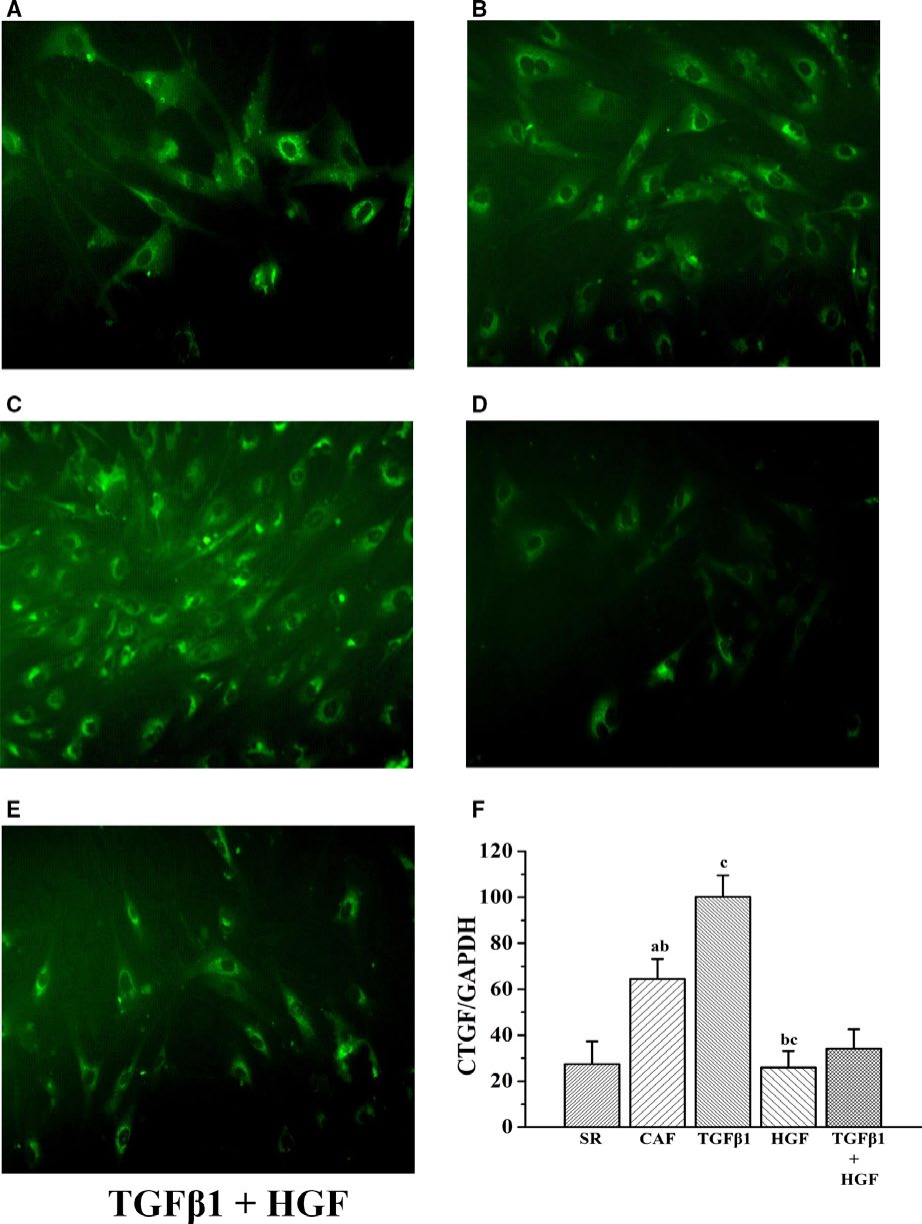
A‐E) Immunofluorescence assay results demonstrating connective tissue growth factor protein expression in each experimental group. F is comparative histograms of CTGF protein expression detected by immunofluorescence in each experimental group (n = 10). In order of appearance (A) the CAF group compared with the SR group (*P* < 0.01) or (B) compared with the TGFβ1 + HGF group (*P* < 0.05); (C) the TGFβ1 group compared with the CAF group (*P* < 0.01); and (B) the HGF group compared with the TGFβ1 + HGF group (*P* < 0.01) and (C) compared with the CAF group (*P* < 0.01)

**Figure 4:**
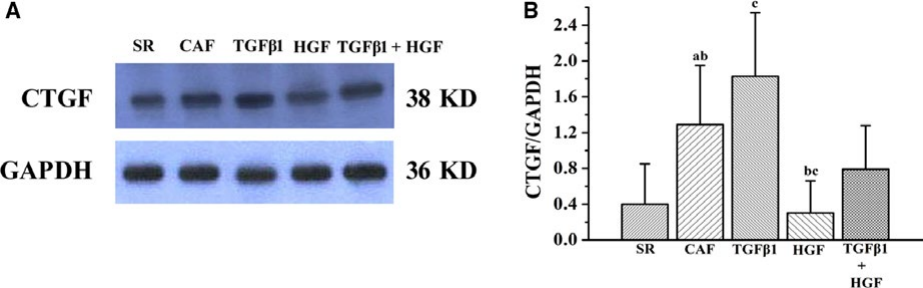
A, Western blot analysis of CTGF protein expression in each experimental group. C is comparative histograms of the CTGF protein expression by Western blot. In order of appearance: (A) the CAF group compared with the SR group (*P* < 0.01) and (B) compared with the TGFβ1 + HGF group (*P* < 0.05); (C) the TGFβ1 group compared with the CAF group (*P* < 0.01); (B) the HGF group compared with the TGFβ1 + HGF group and (C) with the CAF group (*P* < 0.01)

MFb cytoplasmic CTGF fluoresces green using immunofluorescence technology (Figure 3). As measured by immunofluorescence, relative CTGF protein expression levels in the MFbs of the CAF group were significantly increased compared with that of the SR group (*P* < 0.01). CTGF protein expression in the SR, CAF, TGFβ1, HGF and TGFβ1+HGF groups were 27.48 ± 9.84, 64.47 ± 8.64, 100.17 ± 9.34, 25.97 ± 7.14 and 34.21 ± 8.43, respectively (Figure 3). CTGF protein expression levels measured by Western blot assay in the SR, CAF, TGFβ1, HGF and TGFβ1+HGF groups were 0.40 ± 0.45, 1.29 ± 0.66, 1.83 ± 0.71, 0.30 ± 0.36 and 0.79 ± 0.49, respectively (Figure 3). Compared with the CAF group, CTGF protein expression was higher in the TGFβ1 group (*P* < 0.01) and lower in the TGFβ1+HGF (*P* < 0.05) and HGF groups (*P* < 0.01). Compared with the TGFβ1+HGF group, CTGF protein expression was lower in the HGF group (*P* < 0.05) and higher in the TGFβ1 group (*P* < 0.01). The results show consistency between the two methods for CTGF protein expression (Figures 3, 4).

Correlations between the relative CTGF protein levels and LAD or duration of AF episodes in the CAF group were also studied. The results demonstrated that CTGF protein levels were positively correlated with LAD (immunofluorescence: *r* = 0.862, *P* = 0.030, Western blot: *r* = 0.740, *P* = 0.045) and the duration of AF episodes (immunofluorescence: *r* = 0.766, *P* = 0.045, Western blot: *r* = 0.806, *P* = 0.025).

## 4 DISCUSSION

AF is a clinical manifestation comprised of various cardiovascular diseases including RHD^1^ and has become a critical public health problem. Many conditions such as age, hypertension, heart valve disease, coronary heart disease, myocardial infarction, cardiomyopathy and heart failure may lead to the development and progression of AF. Other factors that influence and causes of AF were excluded in this study. No significant differences in gender ratios, age and LVEF among the groups were seen. The pathogenesis of AF remains unclear. It has been confirmed by biopsy and autopsy of AF patients that atrial fibrosis is fundamentally related to the occurrence and development of AF. Atrial fibrosis leads to abnormalities in both AAR and conduction and impairs the electrical conduction velocity, resulting in reentrant circuits in the atrial tissue as well as increased incidence of AF. Fibrosis and increased levels of ECM protein are the basis for persistent and perpetual AF.^23^ However, the mechanism of atrial fibrosis is not fully understood.

More than 50% of the cardiac cells are non‐excitable fibroblasts in healthy hearts. In pathological cardiovascular diseases, fibroblasts can differentiate into α‐SMA‐producing MFbs.^24^ The autonomous and paracrine activities of MFbs are greatly increased, and excessive collagen fibres, cytokines, growth factors and chemokines are synthesized, which affects ECM and collagen production and is involved in the occurrence and development of reactive fibrosis.^25^ Direct coupling of MFbs with cardiomyocytes affects the electrical properties of cardiomyocytes, leading to heterogeneous cardiac conduction.^26^ In addition, atrial wall dilatation promotes the phenotypic transformation of fibroblasts into ECM‐producing MFbs, promoting immune responses and inducing AF‐related AAR by releasing pro‐inflammatory cytokines.^27^ The formation of MF is a process in which multiple factors promote and restrict one another. Atrial fibrosis is not only related to atrial expansion and inflammation, but also to the stimulation of various cytokines. The imbalanced regulation of TGFβ1, HGF and CTGF play an important role in the process. Cardiac fibroblasts are considered to be potentially important target cells. The target cells in this study were atrial fibroblasts of RHD patients. The main mechanisms regarding the interaction among TGFβ1, HGF and CTGF in atrial fibrosis regulation, and the relationship between atrial fibrosis and AF were studied in this work.

TGFβ1 is secreted by cardiomyocytes and fibroblasts. Activated autocrine and paracrine TGFβ pathways induce the differentiation of cardiac fibroblasts into MFbs. It has been established that TGFβ plays a key role in the development of AF‐related cardiac fibrosis.^28^ Overexpression of myocardial TGFβ1 promotes atrial fibrosis, abnormal conduction and increases the incidence of AF. Verheule et al^29^ reported that TGFβ1 expression in transgenic mice selectively led to atrial fibrosis (rather than ventricular fibrosis) and increased AF susceptibility. Xiao et al^30^ showed that the plasma TGFβ1 levels and TGFβ1 expression in atrial tissue were correlated with the degree of left atrial fibrosis and the type of AF, suggesting that TGFβ1 might be involved in the pathogenesis of AF in RHD patients.

Various downstream effects of TGFβ1 that lead to ECM deposition and fibrotic reactions are mediated by CTGF. The specific cellular targets of CTGF are still unclear. It has been found that CTGF activates pro‐fibrotic signalling pathways such as those of Smad, ERK and WNT.^31^, ^32^ TGFβ1 regulates pro‐fibrotic effects by altering Smad protein and CTGF gene expression.^28^ CTGF overexpression is associated with atrial fibrosis in patients with chronic AF.^33^ AF with lowered LVEF is associated with AAR in the AF pig model, which is characterized by increased atrial fibrosis and collagen secretion. These findings support that CTGF plays a key role in the AAR of AF patients.

Our previous studies demonstrated that CTGF and α‐SMA expression in the atrial tissue of AF patients were significantly increased and positively correlated with type I collagen. TGFβ1 expression activated atrial fibroblasts to become MFbs, which then expressed α‐SMA and secreted type I collagen in large amounts, promoting atrial fibrosis in AF patients.^22^ In this work, RT‐PCR results showed that the relative CTGF mRNA levels in MFbs of CAF patients increased significantly. After TGFβ1 intervention, CTGF mRNA expression was significantly up‐regulated, which was consistent with the relative CTGF protein expression detected by the immunofluorescence and Western Blot assays. CTGF Protein expression levels were positively correlated with LAD and the duration of AF episodes in RHD patients. Together with studies in the literature and our previous findings, results of this study suggest that CTGF expression in atrial fibroblasts is up‐regulated in CAF patients and that TGFβ1 further promotes CTGF expression, MFb transformation and activation, and α‐SMA and collagen production, resulting in increased ECM synthesis. The synergistic effect of TGFβ1 and CTGF induces and aggravates atrial fibrosis, causing AAR and provides the mechanisms underlying arrhythmias, which are closely related to AF.

HGF is an anti‐fibrotic factor for the heart with studies that show anti‐fibrotic functions in several animal models and that demonstrate significant alleviation of tissue fibrosis. The mechanism of HGF in MF is unclear; however, it is worth noting that HGF counteracts the effects of TGFβ1 through various mechanisms including inhibition of TGFβ1 secretion, up‐regulation of decorin, inactivation of activated TGFβ1 through the binding of HGF, promotion of myofibroblast apoptosis, down‐regulation of TGFβ1 and interference of Smad signal transduction initiated by TGFβ1. Therefore, these mechanisms could be involved in HGF antagonism of MF in the acute ischemic and dilated cardiomyopathy animal models.^34^ Our previous data^35^ showed that CAF patients had enlarged LAD and increased TGFβ1 secretion. HGF inhibits TGFβ1 production in human atrial fibroblasts and partially inhibits TGFβ1‐induced proliferation.

The reciprocal equilibrium of TGFβ1 and HGF regulates organ fibrosis, whereas HGF could also reverse fibrosis by inhibiting CTGF, downstream of HGF. Galina et al^36^ found that HGF/C‐met reduced collagen secretion and CTGF expression induced by TGFβ1 in lung fibroblasts. Guohua et al^37^ found that HGF inhibited AngII‐induced CTGF the expression in rat fibroblasts and improved myocardial fibrosis. But reports on the direct action of the TGFβ1, CTGF and HGF factor network on human atrial fibroblasts and its relationship to atrial fibrosis have rarely been found.

Our previous study showed that HGF inhibited α‐SMA expression, transformed MFbs, and secreted type I collagen in human atrial fibroblasts.^22^ The results of our study confirmed that MFb CTGF mRNA and protein expression was significantly reduced in the HGF intervention group and that CTGF protein expression was positively correlated with LAD and AF duration in the RHD patients. It was also suggested that CTGF plays a role in atrial remodelling and arrhythmias induced by atrial fibrosis. And thus, with our findings, HGF could directly inhibit α‐SMA and TGFβ1 expression, block expression of factors downstream of TGFβ1, inhibit collagen expression and alleviate atrial fibrosis by inhibiting the transformation of MFbs.

In summary, TGFβ1 positively regulates, and HGF negatively regulates CTGF secretion in atrial myofibroblasts, which, in turn, affects MFb activation, type I collagen and α‐SMA synthesis, and ECM remodelling, thus either promotes or antagonizes atrial fibrosis. HGF likely partially counteracts the atrial profibrotic effect of TGFβ1 by inhibiting CTGF expression in atrial fibroblasts. Further research will focus on the mechanisms of receptor signal transduction among atrial fibroblast cytokines in the atrial fibrosis of AF patients.

Several studies using experimental models have shown that TGFβ1 is a potential anti‐fibrotic target. Caveolin‐1, mitogen‐activated protein kinase 4, and relaxin are negative TGFβ1 regulators that can reverse atrial fibrosis.^38^, ^39^, ^40^ TGFβ1 is an upstream and initiating factor with a wide range of physiological functions; and therefore, its ability to treat AF effectively is limited by its lack of specificity and side effects. Studies have shown that TGFβ1 downstream signalling molecules, such as CTGF, can provide safer and more effective targets for the treatment of these fibrotic diseases.^41^ Therapeutic interventions that inhibit CTGF biological activity have been tested in clinical trials for fibrosis of other organs.^42^ Antibodies against CTGF were shown to partially inhibit TGFβ1‐induced collagen synthesis in fibroblasts. Future research directions will determine whether atrial anti‐CTGF gene transfer can be applied to AF animal models. In these animal models, AF with decreased LVEF is associated with atrial structural remodelling, which is characterized by increased atrial fibrosis and collagen secretion. Profibrotic signalling includes the activation of the TGF‐β1‐Smad‐CTGF pathway and the transdifferentiation of myofibroblasts.^43^ This could provide a basis for future remodelling‐based antiarrhythmic treatments for atrial fibrosis. Recent reports show that MiR‐455 and MiR‐132 reduced myocardial fibrosis by directly inhibiting CTGF,^44^, ^45^ and could be used as new targets for drug therapy.

Based on the literature and experimental results from this study, TGFβ1 and CTGF antibodies could exert anti‐fibrotic effects by blocking TGFβ1 and CTGF. The use of CTGF antagonism, which could be more tissue‐specific than TGFβ1 antagonism, and human recombinant HGF to treat atrial fibrosis merits further research.

## CONFLICT OF INTEREST

The authors confirm that there are no conflicts of interest.
